# Synergistic effects of brain-derived neurotrophic factor (BDNF) and exercise intensity on memory in the adolescent brain: a commentary

**DOI:** 10.1186/s12199-018-0701-8

**Published:** 2018-04-03

**Authors:** Tharmegan Tharmaratnam, Tyler Tabobondung, Taylor Tabobondung, Sally Doherty

**Affiliations:** 1 School of Medicine, Royal College of Surgeons in Ireland-Medical University of Bahrain, Adliya, Bahrain; 2 Brantford General Hospital, Department of Family Medicine, Michael G. DeGroote School of Medicine, McMaster University, Hamilton, ON Canada; 3 Department of Physical and Environmental Sciences, University of Toronto, Toronto, ON Canada; 4 School of Medicine, Royal College of Surgeons in Ireland, Dublin, Ireland

**Keywords:** Brain-derived neurotrophic factor, Memory, Aerobic exercise, Neurogenesis, Exercise intensity, Cognition, Adolescence, Neurogenesis

## Abstract

This commentary highlights the recently published study by Jeon and Ha (Environ Health Prev Med 22:27, 2017) examining the effects of exercise intensity and brain-derived neurotrophic factor (BDNF) on memory in adolescents. This 12-week training study elicited increases in BDNF and improvements in working memory during moderate- and high-intensity exercise, which may have been achieved through improved brain tissue oxygenation, nutrient delivery, and BDNF mRNA expression. These improvements highlight the positive neuroendocrinological effects of BDNF and its role as a potential candidate molecule, as a mediator of synaptic plasticity. In this commentary, we aim to highlight the strengths and potential areas of consideration of Jeon and Ha (Environ Health Prev Med 22:27, 2017). We also offer insight into the clinical implications of this study, such as advocating for exercise in healthy children and as adjunctive therapy in pathological states. This study is promising and further highlights the importance of cardiorespiratory exercise in improving physiological health and cognitive functioning in youth through the phenomenon of neuroplasticity.

## Introduction

Adolescence is a crucial time of brain maturation and alterations in the neuroendocrine milieu. These changes may be augmented synergistically by aerobic exercise and BDNF, a member of the neurotrophin protein family. BDNF operates through a tyrosine kinase b (TrkB) receptor and has been implicated in long-term potentiation, hippocampal neurogenesis, axonal growth, and synaptogenesis [1]. The complex interplay between exercise-induced BDNF production has been shown to induce hippocampal neurogenesis and improvements in working memory [2]. Evidence also exists to suggest that BDNF increases are dependent on exercise intensity, with only high-intensity exercise protocols eliciting BDNF increases [3]. Thus, examining the temporal relationship between exercise intensity and BDNF-mediated cognitive improvements is particularly important during critical periods of development such as adolescence. Additionally, a majority of studies have focused on the adult cohort when investigating this phenomenon.

In a recent training study, Jeon and Ha [4] aimed to elucidate the effects of aerobic exercise of varied intensities on serum BDNF, insulin-like growth factor (IGF-1), cortisol, and working memory of adolescents. The authors recruited a sample of male middle-school students (*N =* 40) to partake in a 12-week treadmill aerobic exercise program comprising of four sessions a week. Participants were randomly assigned to a high intensity (HIEG, 70% oxygen consumption (VO_2_)), moderate (MIEG, 55% VO_2_), low intensity (LIEG, 40% of VO_2_), and a control stretching group (SG). After the training intervention, BDNF expression increased with MIEG (*p* < 0.05) and HIEG (*p* < 0.01), compared to LIEG and SG. Interestingly, there were increases in IGF-1 in only the HIEG and SG, but not MIEG or LIEG. Similar findings were noted for improvements in cortisol levels, with significant reductions in the HIEG group (*p* < 0.05) compared to pre-intervention. The HIEG was also the only group to accrue improvements in working memory (*p* < 0.01) when compared to MIEG, LIEG, or SG [4], which was assessed using the Korean version of the Wechsler Intelligence Scale (K-WISC-111).

This present study lends support to previous work that the magnitude of BDNF increase post-intervention is dependent on exercise intensity in both acute and chronic exercise [5]. A recent meta-analysis demonstrated a dose-response relationship between exercise intensity and peripheral BDNF concentrations in response to acute exercise [1]. Another study showed that high-intensity exercise leads to improved memory via less forgetting of newly learned vocabulary words compared to control group [5]. The magnitude of resting BDNF increases can be further enhanced with a regular exercise program [1], such as in Jeon and Ha [4]. Additionally, Jeon and Ha [4] align with previous work suggesting improvements in executive function and cognition with chronic aerobic exercise [6]. This may suggest a synergistic effect between exercise intensity and magnitude of BDNF production leading to improvements in working memory. Indeed, previous work has shown BDNF levels to be correlated with memory performance [7].

## Strengths and potential areas of consideration

A strength of Jeon and Ha [4] study is the authors accounted for training status of subjects by incorporating a sample size of sedentary individuals. Additionally, the authors standardized participants across the four groups to avoid confounding effects (i.e., body composition was largely homogenous across groups). The study effectively highlights how chronic aerobic high-intensity exercise can lead to improvements in working memory in adolescents. More specifically, this study emphasizes that exercise may in part improve memory during a critical window of development, and this may possibly further attenuate age-related cognitive decline during senescence.

Chronic aerobic exercise is known to induce blood volume expansion, which occurs after 2–3 weeks of exercise, while plasma volume expansion occurs over hours to days [8]. Considering that serum and plasma are components of whole blood, alterations in plasma volume can elicit changes in serum volume. This may result in differing peripheral BDNF concentrations due to exercise-induced angiogenic adaptations, and not merely increased BDNF mRNA expression alone. It is unclear if Jeon and Ha [4] accounted for the cardiovascular adaptations that accompany chronic aerobic exercises and if statistical adjustments were made to account for the variation in BDNF that may result as a function of blood volume relative to increased BDNF gene expression. Additionally, the increased blood volume is also a physiological mechanism which may explain the improvements in VO_2max_ noted in the boys in Jeon and Ha [4], due to improved oxygen delivery because of increased blood volume via training-induced adaptations.

## Issues in the field

While studies have shown increased BDNF levels in the periphery after an exercise intervention [1], it cannot be inferred that central BDNF is also increased in the brain. However, the findings are not equivocal as BDNF has largely been shown to cross the blood-brain barrier (BBB) in murine models. Previous work has shown a relationship between central and peripheral BDNF concentrations [9]. Although murine models have shown that BDNF crosses the BBB bi-directionally, murine models lack validity in this situation, as their BBB differs both anatomically and physiologically when compared to humans. Methodological limitations also persist as there is no standardized method to measure peripheral BDNF, since it exists in both serum and plasma, albeit in differing concentrations. A recent meta-analysis reported no significant differences in studies measuring serum or plasma BDNF, although only a small proportion of studies employed plasma BDNF assessment. Despite the insignificance, studies have shown serum BDNF to be markedly increased relative to plasma BDNF by 200 fold [10]. A majority of studies measure peripheral BDNF in serum, which is essentially stored BDNF in platelets through enhanced mRNA gene expression and transcription, while plasma BDNF represents freely circulating BDNF. Therefore, study variations based on serum or plasma BDNF assessment make it difficult to ascertain definitive conclusions from between-study comparisons. Another methodological limitation is that no biomarker currently exists to measure central, BDNF in living humans, and experimental studies of central BDNF have largely involved murine models.

## Clinical implications

The clinical implications of Jeon and Ha [4] provide further evidence that BDNF is a primary factor in neurogenesis and neuroplasticity [11, 12]. In the future, we hope that further study will provide evidence that implementing aerobic exercise is an evidenced-based and cost-effective adjuvant treatment for those suffering with a variety of psychiatric and neurologic illnesses. The findings from the present study may also be used to advocate for using aerobic exercise as adjunctive therapy for obese children or those with type 2 diabetes mellitus (T2DM). Previous work using functional magnetic resonance imaging (fMRI) has shown atrophy in the subcortical regions of the brain along with temporal and frontal lobes with increased body mass index and a diagnosis of T2DM [13, 14]. These obesity-related declines in brain volume may adversely affect academic performance [14, 15], considering that obesity-mediated changes in brain structure are partly mediated by reduced physical activity [14]. Exercise-induced increases in BDNF have a beneficial role in improving memory and improving the metabolic milieu. BDNF reduces polyphagia, lowers blood glucose, and improves insulin sensitivity and oxidation of glucose [13]. The volume of the hippocampus has also been shown to be greater in physically active children compared to sedentary [13] Thus, the synergistic effects of exercise and BDNF may contribute to improved physiological parameters, academic performance and achievement, and maintenance of a healthy metabolic profile [13, 15]. In healthy children, aerobic exercise during the critical window from childhood to adolescence may thus confer improvements in memory and prevent age-associated cognitive decline.

## Conclusions

Jeon and Ha [4] demonstrated the beneficial effects of exercise intensity on cognition during a 12-week training study in male adolescents with treadmill exercise. Notable improvements were seen in participants’ BDNF levels and working memory (assessed through K-WISC-111) in the HIEG followed by the MIEG. No improvements were seen in the SG and LIEG. This suggests that an exercise intensity of sufficient magnitude may be necessary to induce favorable cognitive improvements. Additionally, this study strengthens the existing literature in regard to the beneficial effects of aerobic exercise for brain health in adolescents. Considering that adolescence is a period of immense neurogenesis, exercise may be a beneficial stimulus to improve memory and scholastic performance during this critical developmental period [15]. What remains unanswered however is the exact physiological mechanism by which aerobic exercise confers improvements in memory. Previous work has suggested that this is mediated by improvements in BDNF in various cortical areas [2], increased cerebral oxygenation, and increased neurotrophin-mediated neurogenesis [1]. Overall, these findings can be used to guide health care providers and encourage them to implement aerobic exercise as an adjuvant therapy for children with psychiatric and neurologic illnesses. Considering that BDNF levels are disordered in those diagnosed with neuropsychiatric disorders and involved in their pathophysiology [12], therapies that affect the concentration of this biomarker in the body need further investigation.

## Abbreviations

BDNF: Brain-derived neurotrophic factor
fMRI: Functional magnetic resonance imaging
HIEG: High-intensity exercise group
IGF-1: Insulin-like growth factor-1
K-WISC-111: Wechsler Intelligence
LIEG: Low-intensity exercise group
MIEG: Moderate-intensity exercise group
SG: Stretching group
T2DM: Type 2 diabetes mellitus
TrKB: Tyrosine kinase receptor b
VO_2_: Oxygen consumption
VO_2max_: Maximal oxygen consumption
